# Identification of two different coagulation phenotypes in people living with HIV with undetectable viral replication

**DOI:** 10.1038/s41598-021-83731-x

**Published:** 2021-02-23

**Authors:** Asbjørn Fink, Andreas Dehlbæk Knudsen, Rebekka Faber Thudium, Jakob Hjorth Von Stemann, Shoaib Afzal, Jens Lundgren, Ditte Marie Kirkegaard-Klitbo, Sisse Rye Ostrowski, Børge G. Nordestgaard, Susanne Dam Nielsen

**Affiliations:** 1 Viro-Immunology Research Unit, Department of Infectious Diseases 8632, Rigshospitalet, Copenhagen University Hospital, University of Copenhagen, Blegdamsvej 9B, 2100 Copenhagen Ø, Denmark; 2 Department of Clinical Immunology 2034, Copenhagen University Hospital, Rigshospitalet, Copenhagen, Denmark; 3 The Copenhagen General Population Study, Department of Clinical Biochemistry, Herlev and Gentofte Hospital, Copenhagen University Hospital, Herlev, Denmark; 4 CHIP, Department of Infectious Diseases 8632, Rigshospitalet, University of Copenhagen, Copenhagen, Denmark; 5 Department of Infectious Diseases, Hvidovre Hospital, University of Copenhagen, Hvidovre, Denmark; 6 Faculty of Health and Medical Sciences, University of Copenhagen, Copenhagen, Denmark

**Keywords:** HIV infections, Thrombosis

## Abstract

Altered coagulation has been reported in people living with HIV (PLWH) with ongoing viral replication and may predispose to cardiovascular diseases. However, less is known about coagulation in PLWH with undetectable viral replication. In a cross-sectional observational study, we investigated whether HIV infection with undetectable viral replication is independently associated with activated partial thromboplastin time (APTT) and coagulation factor II–VII–X concentrations out of reference. Logistic regression analyses were used to assess the association of HIV infection with APTT and coagulation factor II–VII–X, after adjusting for age, sex, smoking status, alcohol consumption, BMI, diabetes and hsCRP. 936 PLWH with undetectable viral replication from the Copenhagen Co-morbidity in HIV infection study (COCOMO-study) and 2955 uninfected controls were included. Higher prevalence of short APTT was found in PLWH compared to controls (13.5% vs. 7.6%, P < 0.001). Furthermore, higher prevalence of low coagulation factor II–VII–X was found in PLWH than in controls (9.6% vs. 7.4%, P = 0.022). HIV was independently associated with short APTT (adjusted odds ratio (aOR) 2.3 (95% CI 1.7–2.9), P < 0.001) and low coagulation factor II–VII–X (aOR 1.4 (95% CI 1.0–1.9), P = 0.046). Few participants among PLWH and controls had both short APTT and low coagulation factor II–VII–X, 2.1% vs. 0.8%, respectively. We found evidence of both procoagulant (short APTT) and anticoagulant (low coagulation factor II–VII–X) alterations in PLWH with undetectable viral replication, and our findings suggest that two different coagulation phenotypes exist in participants with treated HIV infection.

## Introduction

HIV infection is associated with higher prevalence and incidence of cardiovascular diseases (CVD)^1,2^ and thromboses^3,4^. Untreated HIV infection is associated with increased d-dimer^5,6^ reflecting a procoagulant state that may contribute to the increased risk of CVD in people living with HIV (PLWH)^6,7^. In recent guidelines, early initiation of combination antiretroviral therapy (cART) in all PLWH is recommended^8^. Most prior studies of coagulation in treated PLWH have been small or conducted in populations with both untreated and treated participants^9–13^, and no clear consensus regarding whether or not initiation of cART results in normalization of the coagulation system has been reached^14–19^.

Activated partial thromboplastin time (APTT) and coagulation factor II–VII–X are used to assess the contact activation pathway (intrinsic) and the tissue factor activated (extrinsic) pathway of the coagulation cascade, respectively. High APTT and low coagulation factor II–VII–X reflect an anti-coagulant state, while short APTT and high factor II–VII–X concentrations reflect a pro-coagulant state. Lower concentrations of coagulation factor II–VII–X in cART naïve PLWH compared to cART treated PLWH is a consistent finding^6,12^. Prothrombin time (PT), the unstandardized equivalent to INR, that reflects concentrations of coagulation factor II–VII–X has been reported to be both prolonged and short in cART treated PLWH compared to controls^10,20,21^. Furthermore, APTT has been reported to be shorter, prolonged and normal in treated PLWH compared to controls^9,11,13,22^.

We tested the hypothesis that HIV infection is independently associated with levels of APTT and coagulation factor II–VII–X out of reference range. For this purpose, we determined levels of APTT and coagulation factor II–VII–X in PLWH on cART with undetectable viral replication and in uninfected controls.

## Method

### Study design

PLWH were recruited from the Copenhagen Comorbidity in HIV infection (COCOMO) study. The COCOMO study is a longitudinal, observational cohort study aiming to assess the burden of non‐AIDS comorbidity in PLWH. Participants were recruited from out-patient clinics at Departments of Infectious Diseases at University Hospital Copenhagen Rigshospitalet and Hvidovre University Hospital. Inclusion criteria were age ≥ 18 years and a positive HIV test. Between March 2015 and December 2016, 1099 PLWH were enrolled, equivalent to > 40% of all PLWH in the greater Copenhagen area^23^. 985 participants with measurements of APTT and coagulation factor II–VII–X were included in this study and matched 1:3 on birthdate and sex to 2955 HIV uninfected controls from the Copenhagen General Population Study (CGPS). CGPS is an ongoing population study that follows > 100,000 adults from the greater Copenhagen area^24^. 49 PLWH with detectable viral load > 50 copies/mL were excluded. Furthermore, for the analyses of coagulation factor II–VII–X, 45 participants that used anticoagulant medication were excluded. In total, 936 PLWH and 2955 controls matched on age and sex were included in the study.

The study was conducted in accordance to the WMA Declaration of Helsinki and ethical approval was obtained by the Regional Ethics Committee of Copenhagen (COCOMO: H-15017350; CGPS: H-KF-01-144/01). Oral and written informed consent was obtained from all participants.

### Data collection

Information about smoking, medication and current and previous weekly alcohol intake in units, were collected using identical structured questionnaires in COCOMO and CGPS^23^. Information regarding HIV infection was obtained from medical records. Height and weight were measured (Stadiometer and Scale: Soehnle, Nassau, Germany). Body mass index (BMI) was calculated according to international guidelines^25^.

In both COCOMO and CGPS, non-fasting venous blood samples were collected. HbA1c, plasma glucose, high sensitivity C-reactive protein (hsCRP), coagulation factor II–VII–X and APTT were analyzed at the Department of Clinical Biochemistry, Herlev Hospital, Copenhagen (DCB). APTT and coagulation factor II–VII–X were analyzed using ACL TOP (ILS Laboratories Scandinavia, Lillerød, Denmark). The use of coagulation factor II–VII–X as marker of the tissue factor activated pathway instead of the more widely used PT or INR was due to the available coagulation data in the control group.

Diabetes was defined as either use of antidiabetic drugs, non-fasting venous plasma glucose ≥ 11.1 mmol/L and/or HbA1c ≥ 6.5%.

### Definition of outcomes

Altered coagulation was defined as (i) short APTT (APTT < 25 s); (ii) APTT prolonged (APTT > 37 s); and (iii) low coagulation factor II–VII–X (coagulation factor II–VII–X < 63 units/dL). We considered short APTT to reflect a procoagulant state, while prolonged APTT and low coagulation factor II–VII–X were considered to reflect an anticoagulant state. Coagulation factor II–VII–X is not measured above 130 units/dL, therefore there is no above reference for this variable. Reference ranges was provided by DCB.

### Statistical analyses

Demographic differences between PLWH and uninfected controls were assessed using *t* tests and Mann–Whitney *U* comparisons for continuous data, and χ^2^ test for categorical data.

To test for associations between independent variables and values above or below reference range, uni- and multivariable logistic regressions analyses were performed on a dataset including both PLWH and controls for both dependent variables. The multivariable logistic regression model included age (numeric), sex (dichotomous), HIV status (dichotomous), BMI (numeric), smoking (dichotomous), diabetes (dichotomous), alcohol use (numeric) and hs-CRP (numeric).

To determine the association between CD4+ nadir, history of AIDS, hepatitis B and hepatitis C co-infection, and short APTT or low coagulation factor II–VII–X concentrations, logistic regression analyses were performed on a dataset including only PLWH. HIV-related factors were added to the above-mentioned multivariable model one at a time.

A p-value < 0.05 was considered as statistically significant. Statistical analyses were performed using SAS Studio software, version 3.71. Copyright 2020 SAS Institute Inc. SAS and all other SAS Institute Inc. product or service names are registered trademarks or trademarks of SAS Institute Inc., Cary, NC, USA.

## Results

Characteristics of the study population are given in Table 1. Most of participants were males, and the median age was 50 years. There was a higher proportion of current smokers among PLWH, but fewer PLWH were overweight compared to controls. In PLWH, the median (inter quartile range (IQR)) CD4 cell count was 690 cells/µL (530, 890), and 40.7% of PLWH had CD4+ nadir < 200 cells/µL.

**Table 1:** Characteristics of PLWH and uninfected controls.

Characteristics	PLWH (n = 936)	Uninfected controls (n = 2955)
Age, median (IQR)	50.2 (43;58)	50.3 (44;58)
Male sex, n (%)	794 (84.8)	2488 (84.2)
**Smoking, n (%)**		
Never/unknown	348 (37.2)	1506 (51.1)
Former	324 (34.6)	1044 (35.4)
Current	264 (28.2)	400 (13.6)
**Alcohol, units/week, median (IQR)**	84 (24;168)	72 (24;144)
0, n (%)	157 (18.7)	400 (13.6)
1–7 (12–84 g), n (%)	284 (33.9)	1243 (42.3)
> 7 (> 84 g), n (%)	498 (47.4)	1294 (44.1)
Hemoglobin (hgb), mmol/L, mean (95% CI)	9.1 (7.4;10.7)	9.1 (7.7;10.5)
hsCRP, mg/L, median (IQR)	1.2 (0.6;2.5)	0.9 (0.5;1.9)
BMI 18.5 – 24.9, n (%)	481 (52.2)	1020 (35.2)
BMI >25, n (%)	408 (44.3)	1879 (64.3)
Diabetes, n (%)	43 (4.8)	101 (3.4)
Anticoagulant treatment, n (%)	45 (5.3)	89 (3.1)
**Number of altered coagulation factors, n (%)**		
Either low APTT or low coagulation factor 2-7-10	182 (19.4)	239 (8.1)
Both low APTT and low coagulation factor 2-7-10	13 (1.4)	24 (0.8)
**Mode of HIV transmission, n (%)**		
MSM	661 (71.4)	–
Heterosexual	203 (21.9)	–
Injecting drug user	11 (1.2)	–
Other	51 (5.5)	–
Current CD4, cells/µL, median (IQR)	690 (530; 890)	–
Current CD4 < 200 cells/µL, n (%)	16 (1.8)	–
CD4 nadir < 200 cells/µL, n (%)	372 (40.7)	–
Years since human immunodeficiency virus infection, median (IQR)	13.8 (7.1; 21.4)	–
On cART	924 (99.1)	–
Exposed to NRTI, n (%)	880 (94.0)	–
Exposed to NNRTI, n (%)	446 (47.7)	–
Exposed to PI, n (%)	277 (29.6)	–
Exposed to INSTI, n (%)	271 (29.0)	–
Hepatitis B, n (%)	35 (3.8)	–
Hepatitis C, n (%)	44 (5.3)	–

*BMI* Body Mass Index, *cART* combination antiretroviral therapy, *hsCRP* high sensitivity C-reactive protein, *MSM* men who have sex with men, *NRTI* nucleoside/nucleotide reverse transcriptase inhibitors, *NNRTI *non-nucleoside reverse transcriptase inhibitors, *PI* protease inhibitors, *INSTI* integrase inhibitors. Definition of hepatitis B and hepatitis c co-infection was a positive HBsAg and a positive HCV-RNA, respectively.

### Markers of coagulation

Median (IQR) APTT was shorter in PLWH than in controls (27 s. (25, 29) vs. 28.0 s. (26, 30), P < 0.001), and the proportion (95% CI) of PLWH with short APTT was higher than in controls (13.5% (11.5, 15.8) vs. 7.6% (6.6, 8.6), P < 0.001). However, the proportion of PLWH with prolonged APTT was also higher than in controls (2.2% (1.5, 3.4) vs. 1.3% (0.9, 1.8), P = 0.044).

Median (IQR) concentration of coagulation factor II–VII–X was higher in PLWH than in controls (96 units/dL (84, 109) vs. 94 units/dL (83, 106), P = 0.009). However, the proportion of PLWH with low coagulation factor II–VII–X was higher than in controls (9.7% (7.9, 11.7) vs. 7.4% (6.5, 8.4), P = 0.022).

The proportion of participants with both short APTT and low coagulation factor II–VII–X was low in both PLWH and controls (1.4% vs. 0.8%, respectively (P = 0.001). The overlap between the two coagulation phenotypes are illustrated in Venn diagrams as Figure S1 in the supplement.

### Risk factors associated with altered APTT and coagulation factor II–VII–X

Results from multivariable logistic analyses are shown in Table 2. HIV was independently associated with short APTT (aOR 2.2 (95% CI 1.7; 2.9), P =  < 0.001) after adjusting for age, sex, smoking status, alcohol use, BMI, diabetes and hsCRP. Further, higher age, alcohol consumption and BMI > 25 kg/m^2^ was associated with short APTT. In contrast, HIV was not independently associated with prolonged APTT.

**Table 2:** Multivariable logistic regression analyses.

aOR	Low coagulation factor 2–7–10 concentration (< 63 units/dL)		Short APTT (< 25 s.)	
	Adjusted odds ratio 1*	P	Adjusted odds ratio 1*	P
HIV (yes vs no)	1.4 (1.0; 1.8)	0.042	2.2 (1.7; 2.9)	< 0.001
Age (per decade)	1.1 (1.0; 1.3)	0.020	1.1 (1.0; 1.3)	0.044
Male sex (yes vs no)	0.6 (0.5; 0.9)	0.004	0.7 (0.5; 1.0)	0.055
Current smoker (no vs yes)	1.0 (0.7; 1.4)	0.871	0.8 (0.5; 1.0)	0.087
Alcohol, g/week		0.157		0.041
0 (0 g)	Ref.		Ref.	
1–7 (12–84 g)	1.3 (0.9; 1.9)		1.4 (0.9; 2.0)	
8–14 (85–168 g)	1.5 (1.0; 2.2)		1.6 (1.1; 2.4)	
BMI > 25 kg/m^2^ (yes vs no)	1.4 (1.0; 1.9)	0.013	1.6 (1.2; 2.0)	0.003
Diabetes (yes vs no)	1.7 (0.9; 2.8)	0.051	1.2 (0.7; 2.0)	0.560
hsCRP (pr. 10 mg/L)	1.0 (0.8; 1.2)	0.930	0.8 (0.6; 1.2)	0.282

*HIV* human immunodeficiency virus, *BMI* Body Mass Index, *hsCRP* High sensitivity C-reactive protein.

*Adjusted for age, sex, smoking status, alcohol use, BMI, diabetes and hsCRP.

In multivariable analysis, HIV was independently associated with low coagulation factor II–VII–X concentrations (aOR 1.4 (95% CI 1.0; 1.8), P = 0.042). Furthermore, higher age and BMI > 25 kg/m^2^ were associated with low coagulation factor II–VII–X concentrations, while male sex was associated with low coagulation factor II–VII–X concentrations. In a sensitivity analysis, including only PLWH without HCV or HBV, HIV was still independently associated with low coagulation factor II–VII–X (aOR 1.6 (95% CI 1.1; 2.1), P = 0.003) and short APTT (aOR 2.1 (95% CI 1.6; 2.8), P =  < 0.001). To investigate the impact of alcohol on altered coagulation, we performed additional sensitivity analyses excluding PLWH and controls with an alcohol intake > 84 g per week, and found that HIV remained independently associated with low coagulation factor II–VII–X (aOR 1.3 (95% CI 1.0; 1.8), P = 0.044) and short APTT (aOR 2.2 (95% CI 1.7; 2.8), P < 0.001).

In PLWH, we performed a sub-analysis to investigate whether exposure to the different ART classes was associated with either low concentrations of coagulation factor II–VII–X or short APTT. We found that exposure to Non-Nucleoside Reverse Transcriptase Inhibitors (NNRTI) was associated with short APTT (OR 1.8 (95% CI 1.2; 2.7), P = 0.007). None of the other ART classes was associated with low concentrations of coagulation factor II–VII–X or short APTT, data shown in Table S2 in supplement.

CD4+ nadir, history of AIDS, hepatitis B and C co-infection were not associated with altered APTT or coagulation factor II–VII–X. Clinical characteristics of PLWH with and without altered coagulation are shown in Table S1 in the supplement.

## Discussion

In this large cohort of PLWH on cART and with undetectable viral replication and matched uninfected controls, HIV was independently associated with both short APTT and low concentration of coagulation factor II–VII–X suggesting that altered coagulation is a feature of treated HIV infection. A large proportion of PLWH in our cohort had prior AIDS defining events or CD4+ nadir lower than 200 cells/µL and altered coagulation could be due to legacy effects of severe immunodeficiency. However, we did not find any association between altered coagulation and AIDS defining events or low CD4+ nadir to support such a hypothesis.

Short APTT and low concentration of coagulation factor II–VII–X suggest procoagulant and anticoagulant alterations of coagulation, respectively. Since few PLWH had concomitant alterations with both short APTT and low concentration of coagulation factor II–VII–X, HIV may cause two different coagulation phenotypes.

APTT reflects activity of coagulation factor II, VIII, IX, X, XI and XII in the contact activation pathway^26^. We found that HIV was independently associated with short APTT reflecting a procoagulant state. A previous study reported that the contact activation coagulation pathway in PLWH was overactive despite cART and immune reconstitution^22^. In contrast, two studies conducted in African settings found prolonged APTT in PLWH compared to uninfected controls^13,20^. These findings may be explained by inclusion of PLWH with late stage disease and consumption of coagulation factors due to endothelial dysfunction or impaired liver synthesis in the latter studies^13,26,27^. In studies including both treated PLWH and uninfected controls, findings have been inconsistent with regard to possible associations between HIV and APTT^13,20,22^. A recent study found an association between low CD4+ T-cell counts and high viral load and an elevated risk of deep venous thrombosis^4^. We did not find associations between CD4+ T-cell counts or viral load and APTT, probably due to the well-treated nature of our cohort. Importantly, short APTT has been shown to be associated with increased risk of thromboembolic events in uninfected patients^28,29^, and short APTT in treated PLWH may therefore contribute to the observed increased risk of CVD.

We found that HIV was independently associated with low concentration of coagulation factor II–VII–X. Likewise, previous studies have reported a prolonged PT, which reflects low concentrations of coagulation factor II–VII–X, in treated PLWH compared to uninfected controls^10,20,21^. Excessive consumption of coagulation factors may contribute to lower coagulation factor II–VII–X but is less likely since the participants in the present study were clinically well. As factor II–VII–X is synthesized in the liver, our findings could be explained by impaired liver synthesis. In a sensitivity analysis, participants with HBV- or HCV-coinfected were excluded, and we did not find any association between coagulation factor II–VII–X and other markers of liver function including bilirubin and alanin aminotransferase (data not shown). However, minor differences in age, BMI and alcohol consumption between participants with low coagulation factor II–VII–X and participants with no alterations were found and may contribute to the findings. To limit the impact of these differences, models were adjusted for these parameters. Low concentrations of factor II–VII–X may cause an anticoagulant state and lead to elevated risk of bleeding^30–32^.

NNRTI is known to interact with novel anticoagulants and antiplatelet agents, and that may explain our finding^33^.

In the COCOMO cohort, the prevalence of current smokers among PLWH was more than twice as high as in the uninfected controls. We did not find a significant association between either low concentrations of coagulation factors II–VII–X or short APTT and smoking. Smoking is a known major risk factor for CVD, and has multiple effects on hemostasis^34^. Furthermore, there may be an adverse interaction between smoking and HIV on CVD outcomes^35^. Our findings suggest that the increased risk of CVD associated with smoking is not driven by altered coagulation. Prospective investigations of the clinical impact of coagulopathy, including functional coagulation analysis and clinical endpoint evaluations, such as CVD, are warranted.

Limitations to our study included the cross-sectional design, preventing us from determining causality. Matching of PLWH and controls was only done on age and sex, therefore residual confounding regarding lifestyle factors is possible. To assess coagulation using APTT and coagulation factor II–VII–X gives a broad but unspecific picture of the coagulation system, and functional analyses would have provided additional information. Participants in the COCOMO study are mainly Caucasian males, and results may not apply to PLWH of other ethnicities. However, the large number of treated PLWH and controls matched on age and sex from the same geographical area was a major strength. Another strength was information about traditional risk factors for coagulopathy including smoking, alcohol consumption and diabetes, making it possible to adjust for these factors. Finally, all the blood samples were analyzed at the same laboratory limiting inter-laboratory differences.

## Conclusion

Evidence of altered coagulation in well treated PLWH was found, as HIV infection was independently associated with both short APTT and low coagulation factor II–VII–X concentrations. Since few participants had concomitant short APTT and low coagulation factor II–VII–X, our findings suggest that HIV infection is associated with two different coagulation phenotypes, even in well-treated PLWH. Altered coagulation may contribute to increased risk of thromboses or bleeding among PLWH. Prospective investigations of the clinical impact of coagulopathy including functional coagulation analysis are warranted.

## Supplementary Information

Supplementary Information.
