# Supplementary material for: Identification of two different coagulation phenotypes in people living with HIV with undetectable viral replication
Source: Sci Rep. 2021 Feb 23;11:4383. doi: 10.1038/s41598-021-83731-x (PMC7902813; doi:10.1038/s41598-021-83731-x)

Identification of two different coagulation phenotypes in people living with HIV with undetectable viral replication

Running head: Coagulation in HIV

Asbjørn FINK^1^ MD; Andreas Dehlbæk KNUDSEN MD^1^; Rebekka Faber THUDIUM^1^ MD; Jakob Hjorth VON STEMANN^2^ MSc; Shoaib AFZAL^3^ MD PhD DMSc; Jens LUNDGREN^4^ Professor MD DMSc; Ditte Marie KIRKEGAARD-KLITBO^5^ MD; Sisse Rye OSTROWSKI^2^ Ass Professor MD DMSc; Børge G. NORDESTGAARD^3,6^ Professor MD DMSc; Susanne Dam NIELSEN^1^ Professor MD DMSc

^1^Viro-immunology Research Unit, Department of Infectious Diseases 8632, Rigshospitalet, University of Copenhagen, Copenhagen, Denmark; ^2^Department of Clinical Immunology 2034, Copenhagen University Hospital, Rigshospitalet; ^3^The Copenhagen General Population Study, Department of Clinical Biochemistry, Herlev and Gentofte Hospital, Copenhagen University Hospital, Herlev, Denmark; ^4^CHIP, Department of Infectious Diseases 8632, Rigshospitalet, University of Copenhagen, Copenhagen, Denmark ^5^Department of Infectious Diseases, Hvidovre Hospital, University of Copenhagen, Hvidovre, Denmark. ^6^Faculty of Health and Medical Sciences, University of Copenhagen, Denmark

Corresponding author

Susanne Dam Nielsen, MD, DMSc, Professor

Viro-immunology Research Unit, Department of Infectious Diseases 8632

Copenhagen University Hospital, Blegdamsvej 9B; DK-2100 Copenhagen Ø; Denmark

E-mail: [sdn@dadlnet.dk](mailto:sdn@dadlnet.dk); Phone: (+45) 3545 0859, Fax: (+45) 3545 6648

**Table S1: Clinical characteristics of PLWH with and without altered coagulation**

|  | No alterations (741) | Low coagulation factor II-VII-X (72) | Short APTT (110) | Both (13) |
| --- | --- | --- | --- | --- |
| Age, median (IQR) | 50.0  (43.6;57.3) | 51.6^a^  (45.5;60.3) | 52.8^a^ (46.3;61.3) | 51.2 (47;60.1) |
| BMI, median (IQR) | 25.7  (23.4;28.3) | 26.2^a^  (24.1;29.4) | 26.0 (23.4;28.7) | 27.4 (24.6;29.9) |
| Alcohol g/week, median (IQR) | 72  (24;144) | 96^a^  (24;192) | 96  (48;168) | 60  (24;156) |
| Male sex, n (%) | 631 (85.2) | 65 (90.3) | 89 (80.9) | 9 (69.2) |
| ALAT above reference, n (%) | 145 (19.7) | 7 (9.7) | 24 (21.8) | 4 (30.8) |
| CD4, cells/µL, median (IQR) | 690  (520;878) | 700.5  (570;940) | 679.5 (500;900) | 748 (589;1020) |
| CD4 nadir, cells/µL, median (IQR) | 243  (120;356) | 180  (100;341) | 210 (104;312) | 256 (212;410) |
| HCV-RNA positive, n (%) | 38 (5.9) | 3 (4.7) | 3 (3.2) | 0 (0) |
| HBsAg positive, n (%) | 26 (3.6) | 4 (5.6) | 4 (3.7) | 1 (7.7) |

Abbreviations: Low coagulation factor II-VII-X: coagulation factor II-VII-X <63 units/dL; short APTT: APTT <25 seconds; Both: both low coagulation factor II-VII-X and short APTT; BMI: Body Mass Index; ALAT above reference: ALAT >70 U/L for men and >45 U/L for women.

a Significant different from no alterations at the 5% level (Bonferroni-corrected)

b Significant different from Low coagulation factor 2-7-10 at the 5% level (Bonferroni-corrected)

c Significant different from Short APTT at the 5% level (Bonferroni-corrected)

d Significant different from Both Short APTT and Low K2710 at the 5% level (Bonferroni-corrected)

**Table S2: Associations between altered coagulation and different cART classes**

|  | Low coagulation factor II-VII-X concentration (<63 units/dL)  Odds Ratio P | | Short APTT (<25 sec.)  Odds Ratio P | |
| --- | --- | --- | --- | --- |
| NRTI (yes vs no) | 0.7 (0.30;1.8) | .382 | 1.3 (0.6;3.9) | .547 |
| NNRTI (yes vs no) | 1.2 (0.7;1.9) | .460 | 1.8 (1.2;2.7) | .007 |
| PI (yes vs no) | 1.2 (0.7;2.0) | .503 | 0.7 (0.4;1.1) | .108 |
| INSTI (yes vs no) | 0.9 (0.5;1.7) | .732 | 0.7 (0.4;1.1) | .144 |

Abbreviations: NRTI: Nucleoside/Nucleotide Reverse Transcriptase Inhibitors; NNRTI: Non-nucleoside Reverse Transcriptase Inhibitors; PI: Protease Inhibitors; INSTI: Integrase Inhibitors.

**Figure S1: Overlap between the two coagulation phenotypes**


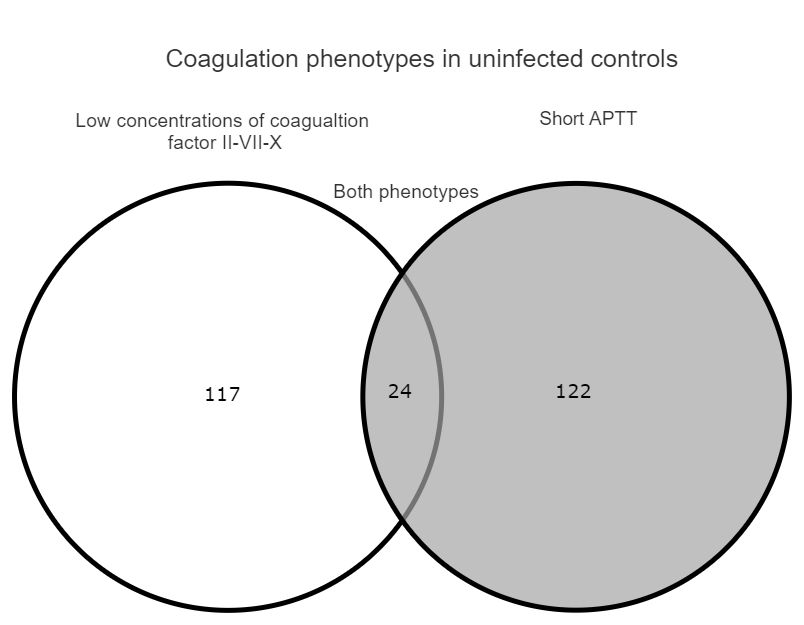


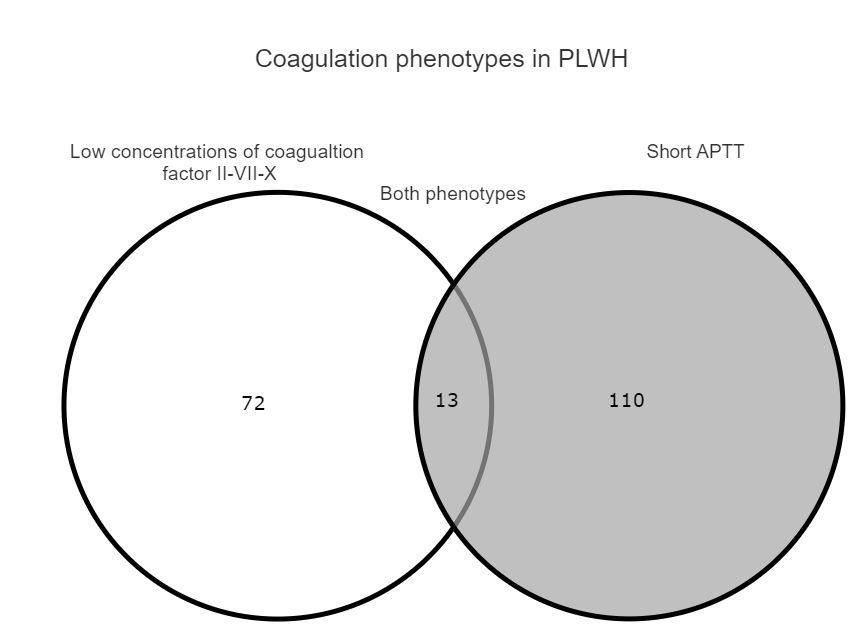

Supplement: Supplementary file 1 — Supplementary Information. [file 41598_2021_83731_MOESM1_ESM.docx]
